# Metallica chair does not spare the sphincter: successful operative reconstruction of an extraperitoneal rectal injury with sphincter involvement

**DOI:** 10.1093/jscr/rjag200

**Published:** 2026-03-29

**Authors:** Clement Rajakumar, Jainika Patel, Khea Tan, Glenn S Parker, Rachel Masia

**Affiliations:** Department of Surgery, Jersey Shore University Medical Center, 1945 Route 33, Neptune, NJ 07753, United States; St. George’s University School of Medicine, St. George’s, Grenada; Department of Surgery, Jersey Shore University Medical Center, 1945 Route 33, Neptune, NJ 07753, United States; Department of Surgery, Jersey Shore University Medical Center, 1945 Route 33, Neptune, NJ 07753, United States; Department of Surgery, Jersey Shore University Medical Center, 1945 Route 33, Neptune, NJ 07753, United States

**Keywords:** extraperitoneal rectal injury, rectal trauma, anal sphincter injury, sphincteroplasty, fecal diversion, penetrating pelvic trauma

## Abstract

Extraperitoneal rectal injuries are uncommon and traditionally managed with fecal diversion, particularly when sphincter involvement is present. We report a case of a 26-year-old male who presented 5 days after a low-energy rectal impalement caused by a collapsed metal chair, with persistent rectal pain, fecal incontinence, and purulent drainage following initial repair at an outside institution. Examination under anesthesia revealed a posterior rectal laceration extending 6 cm from the anal verge, involving ~25% of the rectal circumference with disruption of the internal anal sphincter. After thorough debridement, an overlapping sphincteroplasty was performed without fecal diversion due to viable tissue, minimal contamination, preserved external sphincter function, and hemodynamic stability. The patient recovered uneventfully. This case highlights the feasibility of selective non-diverted management of extraperitoneal rectal trauma with sphincter involvement in carefully selected patients.

## Introduction

Gastrointestinal injuries involving the stomach, small bowel, colon, or rectum may result from either blunt or penetrating trauma, with severity ranging from minor contusions to full-thickness devascularization or perforation. Solid organ injuries are more commonly encountered, while injuries to the colon, and especially the rectum, are relatively rare. Rectal trauma presents unique clinical challenges due to complex pelvic anatomy, the rectum’s retroperitoneal positioning, and the risk of contamination and functional impairment.

The mechanism and energy of the trauma—whether low-energy (e.g. stab wounds) or high-energy (e.g. gunshot wounds)—significantly affect the risk of tissue destruction. High-velocity projectiles, in particular, can cause damage not only via direct impact but also via secondary effects such as cavitation and blast injury. These injuries can compromise local vasculature and lead to delayed tissue necrosis, complicating both diagnosis and management [1].

Rectal injuries are classified by the American Association for the Surgery of Trauma (AAST) Rectum Injury Scale and traditionally managed by the ‘4 Ds’ of rectal trauma: direct repair, diversion, distal rectal washout, and presacral drainage [2, 3]. However, evolving evidence suggests a more selective approach, especially for stable patients with extraperitoneal injuries, limited contamination, and preserved sphincter function.

## Case report

A 26-year-old male with no prior medical or surgical history presented with rectal pain and purulent drainage 5 days after sustaining a traumatic rectal injury at a concert. The patient reported standing on a metal chair that collapsed, impaling his rectum with a rigid pole. At an outside hospital, he underwent contrast-enhanced computed tomography (CT) imaging and primary repair of a rectal laceration. He was discharged with amoxicillin/clavulanic acid and metronidazole.

**Figure 1:**
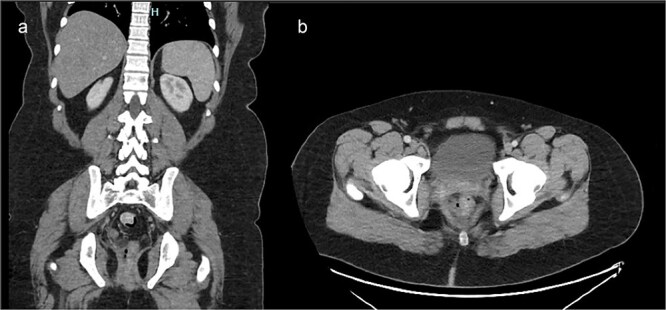
Contrast-enhanced CT of the pelvis. (a) Axial image demonstrating inflammatory stranding and perirectal fluid with foci of extraluminal air adjacent to the posterior rectum. (b) Coronal image showing disruption of the posterior rectal wall with associated soft tissue changes extending into the presacral space.

He re-presented due to ongoing pain limiting his ability to walk, fecal incontinence, and pink-tinged wound drainage. He was hemodynamically stable on presentation. Rectal exam revealed a posterior rectal wound with visible prolene sutures and purulent exudate. Laboratory results showed a mild leukocytosis (white blood cell count 10.7 K/μL), a C-reactive protein of 5.3, and a normal lactic acid. CT imaging demonstrated perirectal inflammatory changes accompanied by foci of adjacent free air, findings concerning for rectal injury (Fig. 1).

**Figure 2:**
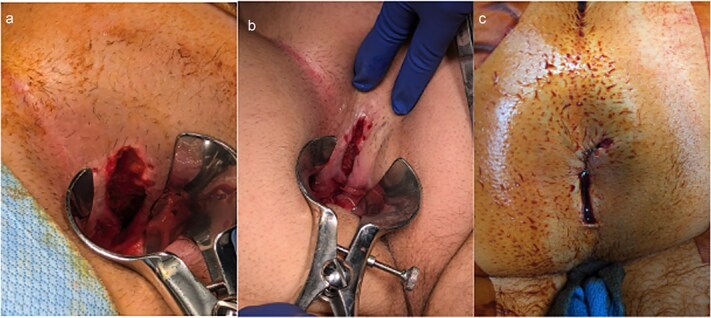
Intraoperative findings and repair of traumatic rectal injury. (a, b) Examination under anesthesia demonstrating the extent of the posterior rectal laceration, involving ~25% of the rectal circumference with disruption of the internal sphincter. (c) Final appearance following overlapping sphincteroplasty with interrupted 2-0 Vicryl sutures, showing restored sphincter continuity and healthy tissue margins.

Given concern for sphincter injury due to ongoing rectal pain and incontinence, the patient was taken to the operating room for an exam under anesthesia. The patient was placed in the prone jackknife position, and on digital rectal exam, sutures could be felt as well as a deeper opening involving the internal sphincter. The prior prolene sutures were removed, and anoscopic evaluation revealed an internal rectal laceration extending 6 cm proximally from the anal verge, involving 25% of the rectal circumference and disrupting the internal sphincter (Fig. 2).

The wound was debrided using a curette, and the wound edges appeared viable without necrosis or infection. The internal sphincter was reapproximated using an overlapping sphincteroplasty technique with interrupted 2-0 Vicryl sutures. The outermost portion of the laceration was left open for drainage due to the infected nature of the case. Given the healthy tissue margins, absence of gross contamination, and preserved external sphincter, fecal diversion was deferred.

Postoperative care included continued antibiotic treatment with piperacillin/tazobactam, multimodal pain control, and maintenance on clear liquids and stool softeners. A Foley catheter was left in place due to high risk for urinary retention. The patient was discharged with oral antibiotics on post-operative day two, and follow-up in the office demonstrated progressive dietary advancement and healing without complications.

## Discussion

Rectal trauma presents diagnostic and management complexities that depend on anatomical location, mechanism of injury, and sphincter involvement.

### Classification and principles of management

Rectal injuries are classified anatomically into:

#### Intraperitoneal injuries

Involve the upper rectum above the peritoneal reflection. These typically require laparotomy and, depending on the extent of contamination, primary repair with or without diverting colostomy. Damage control strategies are indicated for hemodynamically unstable patients, with reoperation once stabilized [3].

#### Extraperitoneal injuries

Occur below the peritoneal reflection, often from penetrating trauma. Historically, management included diversion, distal washout, and presacral drainage. However, modern guidelines from the Eastern Association for the Surgery of Trauma and the Western Trauma Association now recommend a selective approach. In stable patients with minimal contamination, preserved sphincter function, and healthy tissue margins, primary repair without diversion is safe and effective. Distal rectal washout and presacral drainage are no longer routinely recommended, as multiple studies [2, 4, 5] have not demonstrated a mortality benefit and potential increased morbidity. The decision for diversion should be individualized based on the extent of tissue loss, contamination, and associated pelvic injuries.

This patient presented with a low-energy, delayed extraperitoneal injury, which was successfully managed without diversion. Debridement and reapproximation were performed after confirming viable tissue margins and sphincter function.

### Operative strategy and sphincter repair

The key to success in this case was meticulous wound debridement, aggressive irrigation, and precise sphincter repair. Overlapping sphincteroplasty, the gold standard technique for traumatic sphincter injuries, allows reapproximation of the internal sphincter with layered closure, minimizing the risk of incontinence. Interrupted 2-0 Vicryl sutures were used to reduce tension and optimize healing.

Fecal diversion was not performed because of the clean wound bed, intact external sphincter, and stable condition. This decision aligns with selective diversion protocols supported by emerging literature, which show no difference in mortality between diverted and non-diverted patients with appropriately selected injuries [4, 6].

Presacral drainage and distal rectal washout, once common in extraperitoneal rectal trauma, are now discouraged. The EAST guidelines [4] and multiple retrospective studies have found no improvement in mortality or infectious complications with their use. In fact, presacral drainage may increase rates of pelvic sepsis and wound complications. Studies by Ahern *et al.*, and Steele *et al.*, both support omitting these interventions in favor of primary repair or selective diversion when indicated [2, 5]. This shift reflects a broader trend toward evidence-based, minimally invasive management that prioritizes functional outcomes and avoids unnecessary procedures.

## Conclusion

This case demonstrates that extraperitoneal rectal injuries with internal sphincter involvement can be successfully managed without fecal diversion in carefully selected patients. A selective approach emphasizing tissue viability and sphincter preservation may optimize functional outcomes while minimizing morbidity.

## Data Availability

All data relevant to the case are included within the article.
